# Corneal Sensitivity in Keratoconus: A Review of the Literature

**DOI:** 10.1155/2013/683090

**Published:** 2013-10-31

**Authors:** Leopoldo Spadea, Serena Salvatore, Enzo Maria Vingolo

**Affiliations:** Department of Biotechnology and Medical-Surgical Sciences, “La Sapienza” University of Rome, 04100 Latina, Italy

## Abstract

Corneal sensitivity has recently received much attention given the crucial role the corneal nerves play in maintaining normal corneal structure and function. An increased understanding of the corneal sensitivity and dry eye disease in keratoconus, including alterations of the conjunctival cells, may help explain the pathogenesis of this disorder. There is histological evidence of the involvement of corneal nerves in the pathology of keratoconus and it has been suggested that this plays a role in the pathophysiological features and progression of the disease. In this review, the impaired corneal sensitivity found on keratoconus and corneal sensitivity changes after cross-linking performed in patients with keratoconus are reported.

## 1. Introduction

Long-term success of each procedure on the cornea, be it refractive or curative procedures such as keratoplasty, is highly dependent on the integrity of a variety of anatomical structures and physiological factors, such as an intact innervation. The cornea is a highly innervated structure deriving its sensory nerve supply from the trigeminal nerve with the central area being two to three times more sensitive than the peripheral zone. This also reflects the density of nerve supply to the different regions of the cornea [1, 2]. Contact lens wear and intraocular surgical procedures such as cataract extraction can all adversely affect corneal sensitivity [1]. Earlier studies following corneal surgery itself, such as keratotomy, keratectomy, and epikeratophakia, have all confirmed these observations. 

The involvement of corneal nerves in the pathogenesis of keratoconus has not received attention in the past years, and only the prominence and visibility of central corneal nerves have been reported as an early clinical sign of keratoconus [3]. Given the crucial role the corneal nerves play in maintaining normal corneal structure and function, impaired corneal innervation may challenge the ability of the cornea to withstand surgical challenges and thus lead to a significantly increased risk of complications [4]. Nerve status evaluation is crucial to assess the efficacy and long-term effects of the treatments. In fact, there is histological evidence of the involvement of corneal nerves in the pathology of keratoconus, and it has been suggested that this plays a role in the pathophysiological features and progression of the disease.

In this review, we report on the impaired corneal sensitivity found on keratoconus and corneal sensitivity changes after cross-linking performed in patients with keratoconus. 

## 2. Keratoconus

Keratoconus (KC), characterized by a bilateral, noninflammatory corneal ectasia and loss of visual function, is a degenerative disease of the cornea. It affects approximately 1 in 2000 in the general population [5], and its onset is generally at puberty. Incidences of 1 in 600 to 1 in 420 seem to be more in line with current diagnostic tools [6]. Keratoconus manifests itself from a “forme fruste” or subclinical condition, only detectable by computer videokeratoscopy [7] to severe eye disease with the typical biomicroscopy signs: apical protrusion with corneal thinning and scarring, Vogt's striae and Fleisher's ring. KC is progressive in 20% of cases and it was the leading indication for penetrating keratoplasty (21.2%) and anterior lamellar keratoplasty (40.2%) in the United States in 2010 [8].

Classically, onset is at puberty, with progression until the third or fourth decade of life [3], when it usually arrests [7]. Clinically, this corneal ectasia leads to myopia and irregular astigmatism, and in severe cases, ruptures in Descemet's membrane may occur, resulting in corneal edema and scarring [3]. Because of the young age of the patients, keratoconus often has an adverse impact on quality of life [9].

Histopathologic features include changes in corneal collagen structure and organization, alterations of the extracellular matrix, and keratocyte apoptosis and necrosis involving the anterior stroma and Bowman's layer, iron deposition in the epithelial basement membrane, and breaks in the Bowman lamina, which partially explain the biomechanical corneal weakening typical of the disease [7]. Biomechanical changes with increased expression of proteolytic enzymes and decreased concentrations of protease inhibitors, decreased stromal thickness, and modified configuration of collagen lamellae also have been reported among the pathophysiologic mechanisms of the disease [7].

Keratoconus was first described in detail in 1854. Despite intensive clinical and laboratory studies over the last few decades although genetic inheritance and possible linkage with systemic disease have been shown and circumstantial evidence suggests that certain behaviors, such as excessive eye rubbing and contact lens wear, may be associated with the disease, the etiology, and pathogenesis, the causes and possible pathomechanisms for the development of KC remain unclear [7]. 

Tissue degradation in thinning disorders, such as KC, involves the expression of inflammatory mediators, such as proinflammatory cytokines, cell adhesion molecules, and matrix metalloproteinases [10, 11]. Several hypotheses propose genetic, environmental, biomechanical, and biochemical causes and mechanisms [12, 13]. 

Besides penetrating and lamellar keratoplasty [14], hard contact lenses are the major treatment modality for keratoconus. In rare cases, epikeratoplasty, photorefractive keratectomy, or intracorneal rings (which basically are the only transient refractive corrections) can be considered [7]. However, all of these techniques only correct the refractive errors of keratoconus but do not treat the cause underlying the corneal ectasia and therefore cannot stop the progression of keratoconus. An arrest in the progression of KC by contact lenses has been described only in anecdotal reports but has never been confirmed in systematic case series [7]. 

Corneal collagen cross-linking (CXL) is a recent parasurgical technique based on combined use of riboflavin as a photosensitizer and ultraviolet A light of 370 nm. CXL represents the only available treatment directed at the underlying pathology in the keratoconic cornea.

## 3. Corneal Sensitivity

**Figure 1:**
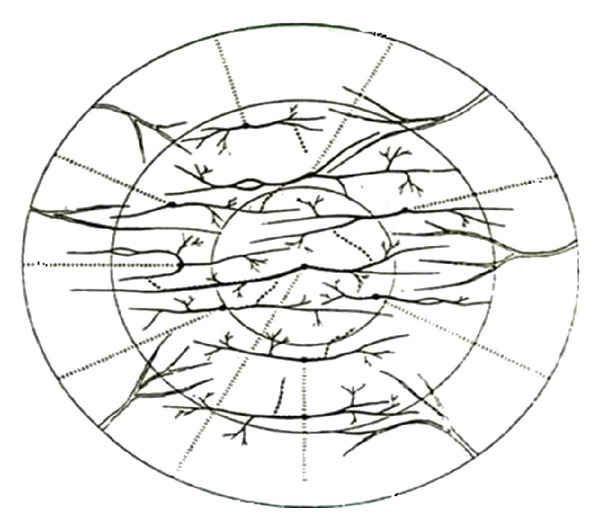
Sensory nerve fibers in the human cornea. The stromal nerves are more evident along the horizontal meridian of the cornea, less along the vertical meridian.

Corneal epithelium is the most densely innervated and exquisitely sensitive surface epithelium of the body. Epithelial nerve density of the cornea is 300–600 times that of the skin [15]. Sensory nerve fibers in the peripheral cornea are myelinated and evident on slit-lamp examination. The very sensitive human cornea contains 30–80 corneal stromal nerve trunks [15]. A gradation appears to exist in the sensitivity of the cornea with sensitivity inversely proportional to the total number of corneal stroma nerve fibers present [15]. Corneal sensitivity is most acute in the central cornea and along the horizontal meridian, least sensitive along the vertical meridian [1] (Figure 1).

Although the entire sensory innervation of the cornea is derived from a relatively small number of neurons, each neuron supports as many as 200–3000 individual corneal nerve endings. The sensory nerves reach the eye through the nasociliary branch of the ophthalmic nerve [15], which branches typically into two long ciliary nerves, one nasal and the other temporal, which course directly to the posterior pole of the eye, and a communicating branch carrying sensory fibers to the ciliary ganglion [1]. Prior to entering the cornea, the nerve bundles traverse the limbus and contribute fibers to the limbal, or pericorneal plexus, a dense, ring-like meshwork of nerve fibers that completely surrounds the peripheral cornea [1]. When penetrating the limbus in the anterior third of the stroma, most of the nerve bundles lose their myelin sheaths, start to divide dichotomously and trichotomously, thereafter bend at right angles, lose their Schwann cell sheath, and penetrate Bowman's lamina to finally enter the epithelium [16, 17]. However, perhaps as many as 30% are finely myelinated A-delta fibers that shed their myelin sheaths within a millimeter or so after entering the cornea [1]. Soon after entering the cornea, each stromal nerve bundle gave rise through repetitive branching to varying numbers of progressively smaller stromal nerves that anastomosed frequently, often at highly acute branch points, to form a moderately dense midstromal plexus. The distal branches of the midstromal nerves often coursed centrally for several millimeters and on occasion crossed the geographic center of the cornea to reach the opposite side. The midstromal plexus in the peripheral stroma occupied roughly the anterior one-half of the stroma while in the central cornea the plexus occupied approximately the anterior one-third [2]. 

Fibers of the midstromal nerve plexus bend horizontally and vertically to form the nerve terminals between the epithelial cells (subepithelial plexus) [1, 16, 17]. The geographical center of the subbasal nerve vortex is located between 2.18 and 2.92 mm from the corneal apex. Near the center of the vortex, the distal segments of the subbasal fibers in some corneas fuse to form an anastomotic network that spirals gently in either a clockwise or counterclockwise direction. In other cases, the subbasal nerves do not form a prominent spiral but end on opposing sides of an imaginary seam-like interface [2]. 

Corneal nerves contain, in varying proportions, the same neuropeptides that are expressed in other ocular nerves. Each corneal fiber population (sensory, sympathetic, and parasympathetic) maintains a distinctive phenotypic signature; however, the chemical coding is complex and most fibers likely express combinations of neuropeptides rather than individual markers. To date, 12 different neuropeptides have been detected by radioimmunoassay or immunohistochemistry in the cornea [1]. 

Corneal sensory nerves protect the cornea from external threats and stimuli by initiating nerve reflex mechanisms [16, 18]. In addition to their important sensory and protective functions, corneal nerves help maintain the functional integrity of the ocular surface by releasing trophic substances (neuropeptides, neurotrophins, and grow factors) that promote corneal epithelial homeostasis and by activating brainstem circuits that stimulate reflex tear production and blinking. Consequently, damage to corneal nerves as the result of surgery, trauma, or disease leads to diminished corneal sensitivity and possible transient or long-term alterations in the functional integrity of the ocular surface [1, 2]. 

Corneal nerves are transected during a variety of corneal and anterior-segment surgical procedures, including refractive surgery, perilimbal incisions performed for cataract surgery, iridectomy and trabeculectomy, and penetrating keratoplasty. Corneal nerves depend for their survival on axoplasmic transport of essential substances from their parent nerve cell bodies in the trigeminal ganglion; thus, surgical procedures that interrupt corneal nerve fibers cause rapid degeneration of the distal axons, decreased corneal sensitivity, and compromised functional integrity of the ocular surface [1, 2]. 

Corneal nerves are capable of regeneration; however, it is a slow, imperfect process and the regeneration that takes place after most corneal surgeries is characterized by reduced nerve density, alterations in nerve architecture, and diminished corneal sensitivity. The more proximally the nerves are cut, the more delayed and incomplete the regeneration process will be. Thus, surgical disruption of the subbasal and subepithelial nerve plexuses produces, in general, less serious and more short-term damage to the corneal innervation than do deep or penetrating incisions that affect major stromal nerve bundles [1]. 

Diseases that are associated with decreased corneal sensitivity in humans include herpetic keratitis, leprosy, diabetes, keratoconjunctivitis sicca, neurotrophic keratitis, and keratoconus.

## 4. Methods of Measurements

**Figure 2:**
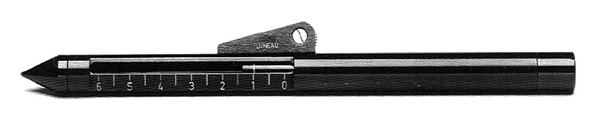
The Cochet-Bonnet esthesiometer.

 The measurement of ocular surface sensitivity is a useful indicator of corneal physiology in corneal disease [18]. Historically, touch sensitivity of the ocular surface has been measured using a von Frey and later Cochet-Bonnet esthesiometer (Figure 2) [19], where a mechanical stimulus is delivered using hair or nylon filaments of variable diameter and length. The stimulus pressure applied is inversely proportional to the filament length, assuming a reproducible amount of bend in the filament.

Regardless of the type of esthesiometer used to measure ocular surface sensitivity, it is key to understand the limitations of the instrument, the stimulus characteristics, normative values, and the repeatability of measurements made. Corneal esthesiometers evaluate corneal sensitivity by measuring the corneal touch threshold (CTT). These instruments usually contain a small-diameter, adjustable, platinum or nylon filament. Pressure for the tip of the filament is able to stimulate circa 100 nerve endings over four to ten corneal epithelial cells [15]. When the corneal threshold pressure to stimulate corneal sensory nerve endings has been reached, the blink reflex will be elicited. The CTT is defined as the pressure at which the majority of touch stimuli cause a blink response. The degree of corneal surface stimulation necessary to cause a blink reflex has been measured with the platinum filament of the Larson-Millidot esthesiometer [20]. 

As stated previously, ocular surface sensitivity has been traditionally measured using the Cochet-Bonnet esthesiometer, which mechanically stimulates the ocular surface with a nylon filament [19]. The deficiencies of this instrument—the most crucial of which are its truncated intensity range and imprecise stimulus application [21]—have led to the development of newer instruments, as the CRCERT-Belmonte esthesiometer [22], which uses a fine jet of gas as a stimulus. The flow, composition, and temperature of this gas can be altered to apply mechanical, chemical, and cooling stimuli to the ocular surface, thus enabling an investigation of the effects of these various stimuli. The instrument incorporates a number of key improvements over earlier air-jet esthesiometers, which permit a more precise application and characterization of the stimulus.

Another esthesiometer, which does not use the nylon filament technique, is the Draeger esthesiometer. The core of the measuring device is a small moving coil galvanometer that is used to produce a force that is transferred on the cornea via a lever that acts as the stimulus tip. This enables the examiner to precisely control the force exerted on the cornea by regulating the intensity of the current. The speed and angle of the test object touching the corneal surface are clearly defined, measurements are completely independent of the humidity of the air, and there is little interobserver variance in the results [23]. The Draeger esthesiometer uses dynamic measurement with an increasing force being exerted while the applicator tip rests on the cornea, thereby covering within one test a large range from complete anesthesia to normal sensitivity. The force that is applied to the cornea can be continuously increased by the observer from 0 to 1000 × 10^−5^ (10^−5 N^ = 1 mp). The endpoint for a positive response is the patient's subjective report of the sensation of the stimulus. The force needed to evoke sensation is digitally recorded.

## 5. Corneal Innervation and Sensitivity in Keratoconus

 The first study that demonstrated impaired corneal sensitivity in keratoconus was carried in 1983 by Millodot and Owens [24]. Secondly, Zabala and Arenas Archila demonstrated the same decrease in corneal sensitivity [25] using the Cochet-Bonnet esthesiometer the authors reported central corneal sensation to be significantly lower than normal in noncontact lens wearing keratoconic eyes, and even lower in keratoconic eyes wearing contact lenses [24, 25]. 

A significant correlation between central corneal sensation and severity of keratoconus has also been reported [24]. Since then a conspicuous number of authors put their effort and attention in the analysis of corneal sensitivity in keratoconus patients. Dogru et al. in 2003 analyzed corneal sensitivity in KC patients and found that 48% of the patients with keratoconus had low corneal sensitivity [26]. The corneal sensitivity was significantly lower in patients with severe keratoconus compared with patients with mild or moderate disease. The authors believed that decreased corneal sensitivity in their series of patients strongly implied corneal epithelial/stromal disease. Mannion et al. noted that subjects with keratoconus who wore contact lenses had significantly lower corneal sensation compared to normal controls who wore contact lenses [27]. Cho et al. in 2013 analyzed corneal sensitivity in patients with asymmetrical keratoconus and found a decrease in corneal sensitivity in all eyes, both in clinical and subclinical KC compared to normal eyes [28]. The authors suggested that decreased corneal sensation was associated with tear deficiency, abnormal impression cytologic results, and thinning of the cornea in KC [28]. 

Recent advances in corneal in vivo confocal imaging have provided new and interesting data on the microstructural alterations of the corneal tissue in keratoconus. Several in vivo confocal microscopy studies consistently have shown a significant derangement in the morphologic and morphometric features of central subbasal and stromal nerves [27, 29–33]. In keeping with these in vivo confocal microscopy findings, Brookes and associates have demonstrated the involvement of corneal nerves in the progression of KC using immunohistochemical analysis [34]. Whether these changes are primary or secondary pathologic manifestations is not clear. However, one theory suggests that accelerated apoptosis and lysis of basal epithelial cells with the release of intracellular proteolytic enzymes are the key triggers of subsequent destructive events involving the underlying corneal tissue, including the nerves in the close vicinity of the affected area [35]. Although this hypothesis could explain the reduction of central subbasal nerve density frequently reported in vivo confocal microscopy investigations, other observations, like loss of the normal subbasal nerve organization [30] and thickening of subbasal [36] and stromal nerves [27, 32], require other explanations. In their study Patel and McGhee [30] were the first to map the architecture of the subbasal nerve plexus in keratoconus and have demonstrated that keratoconus is associated with grossly abnormal subbasal nerve morphology, even in mild keratoconus. The observation that subbasal nerve fiber bundles exhibit the most abnormal configurations at the apex of the cone correlates well with ex vivo studies demonstrating that the greatest destruction of normal corneal architecture occurs at the apex of the cone and that there is a gradient of diminishing damage toward the periphery [34]. In the study by Patel et al. [33] the subbasal nerves qualitatively appeared more tortuous in keratoconic corneas compared to controls. This observation concurs with the results of Mannion et al. [27] who noted a greater range of orientations of subbasal nerve fibers in keratoconic eyes compared with controls. A recent study has demonstrated grossly abnormal subbasal nerve architecture in keratoconus by producing two-dimensional reconstruction maps using in vivo confocal microscopy [30]. At the apex of the cone, a tortuous network of nerve fiber bundles was noted, many of which formed closed loops. At the topographic base of the cone, nerve fiber bundles appeared to follow the contour of the base, with many of the bundles running concentrically in this region [30]. Al-Aqaba et al. reported that thickening of central stromal nerves is a characteristic and a relatively constant feature of keratoconic corneas, in addition to the abnormal stromal nerve tortuosity and overgrowth [37]. Overall, these observations are strongly suggestive of an important role for corneal nerves in the pathophysiology of keratoconus.

## 6. Keratoconus Cross-Linking and Its Impact on Corneal Sensitivity

Collagen cross-linking technique consists of a photopolymerization of stromal collagen fibers induced by the combined action of a photosensitive substance (riboflavin or Vitamin B2) and ultraviolet A (UVA, 370 nm) light that induces corneal stiffening by increasing the number of intrafibrillar and interfibrillar covalent bonds, induces resistance to proteolytic enzymatic degradation such as that from collagenase, and reduces corneal permeability and the formation of large collagen molecular aggregates [38]. Collagen turnover is about 2 to 3 years. Cross-linking freezes stromal collagen, increasing the biomechanical stability of the cornea [38]. 

In addition to keratoconus, cross-linking has been proposed as a therapeutic option for iatrogenic keratectasia in photorefractive keratectomy (PRK) [39] and laser-assisted in situ keratomileusis (LASIK) [38, 40], infectious corneal ulcers [41], pellucid marginal degeneration [42], and bullous keratopathy [43]. 

The first studies in photobiology began in the early 1990s, with attempts to identify biological glues that could be activated by heat or light to increase resistance of stromal collagen. It was discovered that the gluing effect was mediated by an oxidative mechanism associated with hydroxyl radical release. A similar mechanism of hardening and thickening of collagen fibers has been shown in corneal aging and is related to active glycosylation of age-dependent tropocollagen molecules [6]. 

The idea to use this conservative approach to treat keratoconus was conceived in Germany in the 1990s by a research group at Dresden Technical University [43]. The aim was to slow or arrest progression of keratoconus so as to delay or completely avoid perforating keratoplasty. The basis of its use finds support in the evidence that young diabetic patients never have keratoconus, due to the natural cross-linking effect of glucose, which increases corneal resistance in diabetic patients [43]. 

The riboflavin is crucial to the process; in fact, when applied to the anterior corneal stroma, it induces the cross-links and prevents damage to the posterior layers of the cornea and posterior segment of the eye while absorbing the ultraviolet radiation [38, 44]. Contemporary in vivo, ex vivo, and in vitro investigations have revealed significant alterations in the normal architecture and histology of corneal tissues, mainly at the anterior 300 micrometers of the cornea [4, 45]. Despite several clinical and laboratory investigations of the effect of CXL on the corneal microstructure [4], very limited information is available on the status of corneal sensitivity and effect of CXL on corneal innervation in the immediate and long-term posttreatment periods. Most reported studies have used in vivo confocal microscopy to examine corneal nerve changes after CXL [4, 45]. The early effect of standard (following epithelial debridement) and transepithelial CXL on human corneal nerves was studied [46, 47]. According to Al-Aqaba et al. the immediate disappearance of subbasal nerves by confocal microscopy was noted only in corneas that were treated with a standard CXL in which the epithelium was removed. In contrast, they were able to detect these nerves in corneas that were treated with the transepithelial approach. Therefore, it is likely that early disappearance of subbasal nerves is attributable to mechanical scraping of both the epithelium and the subbasal nerves [46]. Mazzotta and associates had speculated that this might be the case but no evidence was provided [48]. 

On histology, however, they could not visualize the subbasal nerves in both the control and treated corneas regardless of the technique. This is an anomaly as the nerves were visible on ex vivo confocal microscopy in the same corneas. This was attributed to the authors to the loss of enzyme activity in the subbasal nerves postmortem [46]. 

There have been contradicting reports on the state of stromal nerves immediately after CXL. While some authors described a complete absence of anterior stromal nerves [4], others have confirmed their visibility in the immediate period after CXL. Al-Aqaba et al.'s results support the latter finding as they were able to demonstrate, by ex vivo confocal microscopy and histology, the presence of stromal nerves within the treatment zone in all the corneas regardless of the treatment protocol. The histochemical detection of deep stromal nerves suggests a better preservation of enzyme reactivity in these nerves compared to the subbasal nerves [46]. 

The reduced corneal sensitivity after CXL reported in previous studies could have a strong relation to the improved contact lens tolerance in keratoconus patients after CXL treatment despite minimal changes in corneal curvature. The author suggested that the tolerance to contact lenses is greatly improved as the sensitivity of the cornea is decreased [46]. Recently, two studies have demonstrated a transient reduction in corneal sensitivity after epithelium-off collagen cross-linking in keratoconic corneas up to 6 months after the procedure [49, 50]. The reduction was greater in the first week after the treatment, and the sensitivity progressively increased during the first 6 months of follow-up. Whether the morphological alteration of the corneal nerve plexus after epithelium-off cross-linking could lead to functional impairment is yet to be investigated. Corneal nerve injury due to deepithelization alone and the increased vulnerability of the corneal nerve plexus in keratoconus may predominantly account for the observed decreased in corneal sensitivity after epithelium-off cross-linking [51]. 

## 7. Conclusion

Corneal sensitivity has recently received much attention given the crucial role the corneal nerves play in maintaining normal corneal structure and function. An increased understanding of the corneal sensitivity in keratoconus may have very useful results to explain the pathogenesis of this disorder and to realize the impact of cross-linking on corneal sensitivity.
